# Indicators for mode of delivery in pregnant women with uteruses scarred by prior caesarean section: a retrospective study of 679 pregnant women

**DOI:** 10.1186/s12884-019-2604-0

**Published:** 2019-11-27

**Authors:** Zhifen Hua, Fadwa El Oualja

**Affiliations:** 1 Department of Obstetrics, Changning Maternity & Infant Health Hospital of Shanghai, No.786, Yuyuan Road, Shanghai, 200050 China; 2 Department of Obstetrics, Moulay Ali Cherif Provincial Hospital, Rashidiya, Morocco

**Keywords:** Caesarean section, Maternal preference, Vaginal delivery, Vaginal birth after caesarean section

## Abstract

**Background:**

The delivery mode for pregnant women with uteruses scarred by prior caesarean section (CS) is a controversial issue, even though the CS rate has risen in the past 20 years. We performed this retrospective study to identify the factors associated with preference for CS or vaginal birth after CS (VBAC).

**Methods:**

Pregnant women (*n* = 679) with scarred uteruses from Moulay Ali Cherif Provincial Hospital, Rashidiya, Morocco, were enrolled. Gestational age, comorbidity, fetal position, gravidity and parity, abnormal amniotic fluid, macrosomia, placenta previa or abruptio, abnormal fetal presentation, premature rupture of fetal membrane with labor failure, poor progression in delivery, and fetal outcomes were recorded.

**Results:**

Out of 679 pregnant women ≥28 gestational weeks, 351 (51.69%) had a preference for CS. Pregnant women showed preference for CS if they were older (95% CI 1.010–1.097), had higher gestational age (95% CI 1.024–1.286), and a shorter period had passed since the last CS (95% CI 0.842–0.992). Prior gravidity (95% CI 0.638–1.166), parity (95% CI 0.453–1.235), vaginal delivery history (95% CI 0.717–1.818), and birth weight (95% CI 1.000–1.001) did not influence CS preference. In comparison with fetal preference, maternal preference was the prior indicator for CS. Correlation analysis showed that pregnant women with longer intervals since the last CS and history of gravidity, parity, and vaginal delivery showed good progress in the first and second stages of vaginal delivery.

**Conclusions:**

We concluded that maternal and gestational age and interval since the last CS promoted CS preference among pregnant women with scarred uteruses.

## Background

One of the most controversial issues in modern obstetrics is how to deliver the second baby of women with uteruses scarred by caesarean section (CS). The CS rate has risen in most countries over the past 20 years. Worldwide, it accounted for 5% of total deliveries in 1970 [1] and increased to over 30% in 2010 [2]. However, this rate ranged from 6.0 to 27.2% in the least and more developed regions in 2014 [2]. In 2008, 64.1% of urban women gave birth by CS in China [3]. The general CS rate was 29% in 2008, and increased to 35% in 2014 [4] and 41.1% in 2016 [5]. This tendency in CS might be due to worldwide socioeconomic change. As previously mentioned, the increased rate of CS has brought about the controversy about the delivery mode for the second baby in cases of uteruses scarred after CS.

It is known that the incidence of uterine rupture (UR) is higher in women with uteruses scarred by CS or laparoscopic surgery for removing salpingectomy (ectopic pregnancy), uterine leiomyomas, and other lesions [6, 7]. Pregnant women with scarred uteruses have higher risk for UR and emergency admission during their pregnancy, and UR during early-stage pregnancy usually ends with pregnancy termination or fetal death [8]. URs during the late stage of pregnancy often end with premature infants with low Apgar scores or transfer to intensive care [9–12]. Despite the increased UR rate due to CS [6], CS shows advantages in newborn and maternal outcomes, in particular for pregnant women with protracted labor, abruptio placentae, placenta previa, fetal embarrassment, or macrosomia, as well as women with abnormal pelvis or poor progress in the first and second stages of labor [1, 13, 14].

Upon introduction of the second-child policy in China in Nov 2013, the controversial issue on how to give birth from a scarred uterus has been put on the table. It has been reported that women with scarred uteruses due to uncompleted CS are often advised to have vaginal birth after CS (VBAC), rather than elective repeat CS (ERCS) [15]. However, this trend reversed from 1996 to 2010 due to worldwide socioeconomic change [16, 17], and then rose to 50% in 2012 [15]. These differences depend on the national healthcare system, clinical guidelines and patient preferences. However, there are no clear guidelines for choosing the second delivery mode after CS.

We performed this retrospective study using data from pregnant women with scarred uteruses at ≥28 gestational weeks to identify the factors associated with CS or VBAC preferences. Factors including maternal age, gestational age, interval since the last CS delivery, and number of vaginal deliveries prior to and after the last CS, as well as the episodes related to childbirth, were recorded and analyzed. We believe that this summary will facilitate decision-making regarding vaginal vs. CS delivery for women with prior CS.

### Materials and methods

#### Ethical approval and data protection

The de-identified data collected were obtained directly from this hospital’s database. Consent was obtained from patients before data collection. The Moulay Ali Cherif Provincial Hospital Ethics Committee ruled that ethical approval was not needed for the study. A confidentiality statement of data protection was completed with the data protection officer at the Moulay Ali Cherif Provincial Hospital.

#### Patient profile

This retrospective study was based on data from pregnant women with scarred uteruses admitted to Moulay Ali Cherif Provincial Hospital, Rashidiya, Morocco, between October 2016 and September 2018.

Women were included in our study if they met the following inclusion criteria: (1) age 19 or over, (2) with history of single-scar uterus, and (3) at ≥28 gestational weeks. The womens’ medical histories (gynecology and obstetrics), surgical indications for the last CS, number of vaginal deliveries prior to and after the last CS, as well as the interval between the last CS and this pregnancy (years), were recorded. The episodes related to childbirth captured the number of gravidity and parity, delivery method, and outcome.

Upon admission, we recorded the pregnant womens’ demographic characteristics, including gestational age (≥ 28 weeks), comorbidity history (including chronic hypertension, diabetes and gestational diabetes, and hypertension), electronic fetal heart monitoring, and fetal position, number, and presentation. Pregnant women with opening or opened cervical canals were prepared for VBAC. Pregnant women with indications for ERCS (pre-labor CSs) or intrapartum CS were not indicated, but the indications for CS were presented, including multifetation (≥ 2); suspected UR (with indications of abdominal pain, preeclampsia or eclampsia, and vaginal bleeding); abnormal amniotic fluid (pollution or less); macrosomia (evaluated over birthweight); placenta previa or abruptio placentae; abnormal fetal presentation; premature rupture of fetal membrane with labor failure; comorbidity (as mentioned above); and protracted active phase dilatation, protracted descent pattern, and prolonged latent phase of labor.

All episodes related to childbirth, including Apgar score (0–5 min), amount of bleeding during delivery (mL), and first- and second-stage labor duration (hour and minute; only for vaginal birth) were captured.

#### Statistical analysis

Pregnant women with CS and vaginal delivery were assigned to two groups accordingly. SPSS 22.0 software was used for statistical analysis. The difference in normally distributed data (age) between groups was analyzed using the unpaired t-test and that of abnormally distributed data (including gravidity and parity numbers, gestational age, and interval from the last CS) was analyzed using the Mann-Whitney U test. Differences in descriptive data (prior vaginal delivery, maternal demographics, indications for CS, and fetal outcomes) between the two groups were analyzed using the χ^2^ test with or without weighted case (frequency). To evaluate the correlation between maternal demographics, CS indicators, and fetal outcomes, we calculated the Spearman correlation coefficients. The 95% confidence intervals (95% CI) were analyzed. Logistic regression analysis was performed to identify indicators for CS preference. For all analyses, *p* < 0.05 was defined as statistical difference.

### Results

#### Demographic characteristics of participants in the CS and VBAC groups

Out of the 679 pregnant women ≥28 gestational weeks, 351 women were assigned to CS (51.69%) and the other 328 women were assigned to VBAC (48.31%). There was no difference in age between the two groups (29.68 ± 6.04 vs. 29.70 ± 5.36, *p* = 0.979, Table 1). Women in the CS group showed significantly lower gravidity number, prior vaginal delivery number, shorter interval since the last CS, higher parity frequency, and older gestational age compared to women assigned to the vaginal delivery group (*p* < 0.01 for all). There were no significant differences in CS preference among ages (*p* = 0.169; χ^2^ test), interval since the last CS (*p* = 0.535), and newborn Apgar score (*p* = 0.222) between the women assigned to the CS and VBAC groups (Table 2).

**Table 1:** Baseline characteristics of pregnant women in the CS and VBAC groups

	VBAC (*n* = 328)	CS (*n* = 351)	p
Age (year, mean ± SD)	29.68 ± 6.04	29.70 ± 5.36	0.979 ^a^
Gravidity (times, mean ± SD)	2.86 ± 1.32	2.53 ± 0.96	0.000^b^
Parity (mean ± SD)	1.34 ± 0.79	1.59 ± 1.01	0.000^b^
Gestational age (weeks, mean ± SD)	39.54 ± 1.72	39.92 ± 1.55	0.0027^b^
Prior vaginal delivery (Yes/No)	60 (18.29%)	23 (6.55%)	0.0001^c^
Interval since the last CS (year)	4.79 ± 3.11	3.97 ± 2.21	0.002^b^

*CS* cesarean section, *VBAC* vaginal birth after CS

^a^unpaired t-test; ^b^ Mann-Whitney U test; ^c^ χ^2^ test

**Table 2:** Differences between factors for the CS and VBAC groups

	Grouping	CS (351)	VBAC (328)	P
Maternal age				
	< 24	42 (11.96%)	54 (16.46%)	0.169^c^
	24–34	234 (66.67%)	199 (60.67%)	
	> 34	75 (21.37)	75 (22.86%)	
Interval since the last CS				
	< 2 years	24 (6.84%)	22 (6.71%)	0.535 ^c^
	≥2 years	327 (93.16%)	306 (93.29%)	
Fetal outcome (Apgar score)				
	0	6 (1.71%)	3 (0.91%)	0.222 ^c^
	1–3 score	0	2 (0.61%)	
	4–7 score	3 (0.85%)	5 (1.52%)	
	8–10 score	342 (97.44%)	318 (96.96%)	
Birth weight				
	< 2500 g	10 (2.85%)	14 (4.27%)	0.043 ^c^
	2500-4000 g	288 (82.05%)	284 (86.58%)	
	≥4000 g	53 (15.10%)	30 (9.15%)	

^c^ χ^2^ test, *CS* cesarean section, *VBAC* vaginal birth after CS

Most women (60.67% in the VBAC group and 66.67% in the CS group) were 24–34 years old; approximately 22% of women were older than 34 years in the two groups. More than 93% women (93.29% in the VBAC group and 93.16% in the CS group) had over a 2-year interval since the last CS, suggesting good health consciousness. Most fetuses (approximately 97%) showed good outcomes, with Apgar score above 8′-8′. Pregnant women with newborns weighing over 4000 g had CS selection preference (*p* = 0.043, Table 2).

#### Indicators for CS preference

The factors that might impact maternal preference for CS were analyzed using logistic regression analysis. Maternal age (β = 0.043, 95% CI 1.010–1.097, *p* = 0.010), gestational age (β = 0.138, 95% CI 1.024–1.286, *p* = 0.018), and interval since the last CS (β = − 0.090, 95% CI 0.842~0.992, *p* = 0.031) were indicators for CS (Table 3). Higher maternal and gestational ages and shorter interval since the last CS were shown to promote maternal preference for CS. The history of gravidity and parity, vaginal delivery, and high birth weight were not indicators for CS preference (*p* > 0.05; Table 3).

**Table 3:** Identification of factors associated with selection of CS by logistic regression analysis

Indicators	β	OR	95% CI	P
Maternal age (years)	0.043	1.044	1.010–1.097	0.010
Gravidity (times)	−0.148	0.863	0.638–1.166	0.336
Parity (times)	−0.291	0.748	0.453–1.235	0.256
Gestational age (weeks)	0.138	1.148	1.024–1.286	0.018
Interval from the last CS	−0.090	0.914	0.842–0.992	0.031
Prior vaginal delivery (times)	0.133	1.142	0.717–1.818	0.577
Birth weight	0.000	1.000	1.000–1.001	0.135

*CS* cesarean section, *OR* odds ratio

Out of 351 women in the CS group, 144 participants (41.03%) chose CS delivery facing certain fetal conditions, including postmature birth (*n* = 57, 39.58%), followed by abnormal amniotic fluid (*n* = 37, 25.69%; Table 4). Of the risk factors in pregnant women, abnormal pelvis (*n* = 58, 28.02%), poor active phase (including protracted active phase dilatation, protracted descent pattern and prolonged latent phase of labor; *n* = 50, 24.15%), and malpresentation (*n* = 28, 13.53%) were the first three reasons for CS preference. Of the 16 participants with suspected UR, 7 (43.75%) had hemorrhoea (> 500 ml).

**Table 4:** Indicators for CS selection

Indicators	n = 351	P
Maternal preference	*n* = 207 (58.97%)	0.000^c^
Comorbidity	21	
Abnormal pelvic	58	
Abnormal AP	50	
Suspected UR	16	
Short interval (< 2 years)	10	
Abruptio placentae/PROM	24	
Malpresentation	28	
Fetal preference	*n* = 144 (41.03%)	
Postmature birth	57	
Macrosomia	22	
Abnormal AF	37	
Twins of multiple birth	9	
Embarrassment	10	
Others	9	

^c^ χ^2^ test; CS, cesarean section; PROM, premature rupture of membrane; Abnormal AF (amniotic fluid) includes less amniotic fluid and amniotic fluid pollution; Abnormal AP (active phase) includes protracted active phase dilatation, protracted descent pattern and prolonged latent phase of labor. Comorbidity includes hypertension, diabetes and preeclampsia or eclampsia; UR, uterus rupture; Malpresentation includes breech presentation and transverse presentation; Other reasons for fetal conditions include history of fetal death/abnormal heart rate

#### Risk factors associated with outcomes of CS delivery

Next, we analyzed the correlation between episodes related to CS, including suspected UR, abnormal amniotic fluid, birthweight, maternal age, gestational age, retarded birth, abnormal fetal presentation, comorbidity (including hypertension, diabetes, and preeclampsia or eclampsia), and Apgar score. Spearman correlation analysis showed that the presence of birth defects was negatively correlated with amniotic fluid abnormity (β = − 0.172, *p* < 0.01) and UR (β = − 0.167, *p* < 0.01) and positively correlated with birthweight (β = 0.170, *p* < 0.01; Table 5). Moreover, suspected UR showed negative correlations with gestational age (β = − 0.231, *p* < 0.01), abnormal active phase (β = − 0.145, *p* < 0.05), and birthweight (β = − 0.197, *p* < 0.01). Women with abnormal fetal presentation often had lower gestational age (β = − 0.110, *p* < 0.05), abnormal amniotic fluid (β = − 0.110, *p* < 0.05), and abnormal active phase (β = − 0.126, *p* < 0.05). Increased gestational age was only correlated with incidence of macrosomia (β = 0.307, *p* < 0.01), and abnormal amniotic fluid was negatively associated with fetal Apgar scores (β = − 0.268, *p* < 0.01; Table 5).

**Table 5:** Spearman correlation coefficients between characteristics related to CS

	M. age (Years)	G. age (weeks)	Retarded birth	Abnormal AF	Abnormal AP	Comorbidity	Suspected UR	Abnormal P	Weight (g)	Apgar score
M. age (Years)	1.000	−0.026	−0.045	− 0.006	− 0.005	0.043	−0.034	0.006	0.050	0.020
G. age (weeks)	− 0.026	1.000	0.571^**^	0.069	−0.130^*^	− 0.086	− 0.231^**^	−0.110^*^	0.307^**^	0.002
Retarded birth	−0.045	0.571^**^	1.000	−0.172^**^	− 0.197^**^	− 0.108	− 0.167^**^	− 0.089	0.170^**^	0.097
Abnormal AF	−0.006	0.069	−0.172^**^	1.000	−0.149^**^	− 0.082	− 0.096	−0.110^*^	0.094	−0.268^**^
Abnormal AP	−0.005	−0.130^*^	− 0.197^**^	−0.149^**^	1.000	−0.093	− 0.145^**^	− 0.126^*^	− 0.098	0.084
Comorbidity	0.043	−0.086	− 0.108	− 0.082	− 0.093	1.000	−0.079	− 0.069	− 0.079	− 0.028
Suspected UR	−0.034	− 0.231^**^	− 0.167^**^	− 0.096	− 0.145^**^	− 0.079	1.000	− 0.072	− 0.191^**^	0.071
Malp.	0.006	−0.110^*^	−0.089	− 0.110^*^	− 0.126^*^	− 0.069	−0.072	1.000	0.006	0.062
Weight (g)	0.050	0.307^**^	0.170^**^	0.094	−0.098	− 0.079	− 0.191^**^	0.006	1.000	0.067
Apgar score	0.020	0.002	0.097	−0.268^**^	0.084	−0.028	0.071	0.062	0.067	1.000

G. age, gestational age; M. age, maternal age; AF, amniotic fluid, included less and pollution; AP, active phase. Abnormal AP includes protracted active phase dilatation, protracted descent pattern and prolonged latent phase of labor. Comorbidity includes hypertension, diabetes and preeclampsia or eclampsia; UR, uterus rupture; Malp (malpresentation), abnormal presentation, includes breech presentation and transverse presentation; *, *p* < 0.05; ** *p* < 0.01

#### Factors associated with outcomes of vaginal delivery

Spearman correlation analysis was also performed to identify the correlations between factors associated with VBAC (Table 6). We found that the duration of first-stage and second-stage deliveries was significantly correlated (β = 0.452, *p* < 0.01). The delivery of macrosomia might increase duration of second-stage deliveries (β = 0.340, *p* < 0.01). Pregnant women with higher number of vaginal deliveries and longer intervals since the last CS showed shorter delivery duration (*p* < 0.01). In addition, we noted that older women showed shorter duration of first-stage (β = − 0.153, *p* < 0.05) and second-stage (β = − 0.245, *p* < 0.01) deliveries. Higher number of parity correlated with shorter delivery duration (β = − 0.307, *p* < 0.01; Table 6).

**Table 6:** Spearman correlation coefficients between factors associated with outcomes of VBAC

	D. duration (min)	O. time (h)	M. age (year)	Weight (g)	After VD	Prior to VD	Interval (year)	G. age (weeks)	Gravidity	Parity
D. duration (min)	1.000	0.452^**^	−0.245^**^	0.340^**^	−0.228^**^	−0.199^**^	− 0.163^*^	0.093	− 0.225^**^	−0.307^**^
O. time (h)	0.452^**^	1.000	−0.153^*^	0.115	−0.094	−0.055	− 0.149^*^	0.082	− 0.141	−0.100
M. age (year)	−0.245^**^	−0.153^*^	1.000	0.004	0.377^**^	0.386^**^	0.422^**^	−0.194^**^	0.503^**^	0.560^**^
Weight (g)	0.340^**^	0.115	0.004	1.000	0.089	0.072	0.038	0.168^*^	0.131	0.100
After VD	−0.228^**^	−0.094	0.377^**^	0.089	1.000	−0.064	0.498^**^	−0.054	0.455^**^	0.607^**^
Prior to VD	−0.199^**^	−0.055	0.386^**^	0.072	−0.064	1.000	0.119	−0.087	0.584^**^	0.704^**^
Interval (year)	−0.163^*^	−0.149^*^	0.422^**^	0.038	0.498^**^	0.119	1.000	−0.069	0.343^**^	0.411^**^
G. age (weeks)	0.093	0.082	−0.194^**^	0.168^*^	−0.054	−0.087	− 0.069	1.000	−.120	− 0.118
Gravidity	−0.225^**^	− 0.141	0.503^**^	0.131	0.455^**^	0.584^**^	0.343^**^	−0.120	1.000	0.786^**^
Parity	−0.307^**^	−0.100	0.560^**^	0.100	0.607^**^	0.704^**^	0.411^**^	−0.118	0.786^**^	1.000

Prior to and after VD number, the number of vaginal deliveries (VD) prior to and after the last cesarean section (CS). G. age, gestational age; M. age, maternal age; VBAC, vaginal birth after CS

Gravidity and parity, the number of gravidity and parity prior to this vaginal delivery. D. duration, the duration of the second stage delivery. O. time, opening time, the duration of the first stage of delivery (time spent on opening cervical canal). *, *p* < 0.05; ** *p* < 0.01

## Discussion

Our study confirmed that most pregnant women with scarred uteruses chose CS delivery (51.69%), which was consistent with the worldwide trend. Of 679 CS deliveries, 41.03% were compelled by fetal conditions, including breech presentation, transverse presentation, reduced amniotic fluid, and amniotic fluid pollution. Of the other cases, 58.97% were caused by maternal conditions, 28.02% had abnormal pelvic, 24.15% developed abnormal active phase (protracted active phase dilatation, protracted descent pattern, and prolonged latent phase of labor), and 13.53% showed malpresentation during vaginal delivery. This was in accordance with the generally reported reasons for CS delivery, including protracted labor, abruptio placentae, previous CS, eclampsia, placenta previa, and malpresentation [1].

We determined that pregnant women with newborns weighing over 4000 g had CS preference, suggesting that macrosomia was an indicator for CS. Guo et al. showed that there was significantly lower neonatal birth weight between women in the VBAC group and women who failed the trial of labor after caesarean section (TOLAC) [18]. That was true for the women both with successful and failed TOLAC [19]. They also showed that vaginal delivery history and birth weight (< 3300 g) were independent factors for VBAC or successful TOLAC [18, 19]. However, there is controversy about the choice for macrosomia delivery in the general population. Menticoglou et al. [20] showed that most macrosomia (78.7%) were delivered via labor in Melbourne, Australia, with good outcomes, based on general cohort not considering scarred uterus.

Through our analysis, we determined that age was not a causality for CS preference in pregnant women (95% 1.010–1.097). Actually, some studies showed that advanced maternal age correlated with failed TOLAC [19], and maternal age < 35 years correlated with trial of labor [21]. This was different from the correlation between older maternal age and VBAC preference reported by Guo et al. [18]. However, Minsart et al. showed that Chinese women had a higher vaginal birth rate after CS compared with North American and Australian women [21]. This showed that the rate of vaginal birth after CS is different between races regardless of the age effect [21].

Sudhof et al. [22] showed that spontaneous labor onset was an indicator for choosing vaginal delivery. However, many parous women with or without spontaneous labor onset are more likely to have a cesarean birth [23]. This is a consequence of the fear of vaginal pain among many women. Out of 679 pregnant women, we found that older participants showed shorter duration of delivery (both in the first and second stages). This fact is largely due to the higher number of previous vaginal delivery among older women. We also found that the age of pregnant women was positively correlated with shorter gestational age especially in women in the VBAC group, which might indirectly reduce the incidence of macrosomia. Similarly, a negative correlation between advanced maternal age and incidence of macrosomia was reported by Lin et al. [24]. Advanced maternal age and higher birth weight were reported to be correlated with higher rate of failed TOLAC [19]. However, advanced maternal age (> 30 years) may be associated with higher risk of small for gestational age < 10th percentile [25], while women aged 20 to 29 years old had low risk of small for gestational age < 10th percentile.

We found that history of prior vaginal delivery was not an indicator for the selection of vaginal delivery mode or that there was even a correlation between them. This was not in accordance with the results obtained by other investigators [26] who have shown that prior history of vaginal delivery was a major reason for vaginal delivery preference. Some investigations show that both maternal age and parity number are associated with increased incidence of UR [6] and placenta previa [27, 28]. However, our study did not present the correlation between maternal age and incidence of suspected UR.

Women with a longer interval since the last CS had a preference for vaginal delivery. This was in accordance with the results reported by Seffah and Adu-Bonsaffoh [19], who showed that a short inter-pregnancy interval was related to failed TOLAC. We noted that more than 93% women (93.29% in the VBAC group and 93.16% in the CS group) had over a 2-year interval since the last CS, suggesting good health consciousness in most pregnant women. It has been reported that the mature stage for scarring is approximately 2 years [29, 30], and the following pregnancies usually show good outcomes with low tendency toward UR. Pregnant women with earlier healing and hospital discharge had a preference for vaginal delivery [26]. Two of the 16 women with suspected UR in our study had shorter intervals (< 2 years; 12.5%). However, 15 of 16 participants showed good outcomes with high Apgar scores (10–10) due to CS. These results suggested that CS was effective for improving the outcomes of pregnant women with UR.

## Conclusions

In summary, we concluded that increased maternal and gestational ages and a shorter interval since the last CS were indicators for CS delivery. There was a negative correlation between maternal and gestational age, which might reduce the frequency of macrosomia. Both CS and VBAC are viable options for childbirth, with comparable fetal outcomes. The selection of repeat CS delivery after CS may not depend on maternal age and gestational age, but may be associated with adequate patient education.

## Data Availability

The datasets generated and analyzed during the current study are not publicly available due to institutional confidentiality but are available from the corresponding author on reasonable request.

## Abbreviations

CS: Cesarean section
ERCS: Elective repeat CS
UR: Uterus rupture
VBAC: Vaginal birth after CS
